# Synergistic Effects of Hydroxychloride and Organic Zinc on Performance, Carcass Characteristics, Liver and Tibia Mineral Profiles of Broiler Chickens

**DOI:** 10.1007/s12011-024-04385-0

**Published:** 2024-09-23

**Authors:** Vahideh Sadr, Hoai Thi Thanh Nguyen, Lane Pineda, Yanming Han, Mehdi Toghyani

**Affiliations:** 1 School of Life and Environmental Sciences, Faculty of Science, The University of Sydney, Sydney, NSW 2006 Australia; 2 Poultry Research Foundation, The University of Sydney, Camden, NSW 2570 Australia; 3 Department of Animal Science, School of Environmental and Rural Science, University of New England, Armidale, NSW 2351 Australia; 4 Trouw Nutrition, Amersfoort, Netherlands

**Keywords:** Supplemental zinc, Inorganic, Organic, Hydroxychloride, Broiler chickens

## Abstract

This study investigated the potential synergism between hydroxychloride and organic zinc (Zn) at different levels on performance and carcass characteristics of broiler chickens. There were seven experimental diets including a negative control diet without any supplemental Zn, and six diets with 80 mg/kg added Zn in the forms of ZnSO_4_, hydroxychloride Zn (HCZ) and organic Zn (ORZ), and a combination of HCZ and ORZ at 40 mg/kg HCZ + 40 mg/kg ORZ (HCZ40-ORZ40), 55 mg/kg HCZ + 25 mg/kg ORZ (HCZ55-ORZ25), and 70 mg/kg HCZ + 10 mg/kg ORZ (HCZ70-ORZ10). Each diet was replicated eight times with 17 chicks per replicate. On day 35, HCZ70-ORZ10 and HCZ40-ORZ40 diets resulted in the highest body weights (*P* < 0.05). Throughout the entire production period (1–35 days), all HCZ and ORZ diets significantly improved feed conversion ratio (FCR) compared to PC (*P* < 0.05), with HCZ40-ORZ40 showing the lowest FCR. Breast meat yield was lower in NC-fed birds, while HCZ80 and HCZ70-ORZ10 groups had the lowest abdominal fat weight (*P* < 0.05). Liver and gizzard weight, tibia breaking strength, and ash percentage, footpad dermatitis, and hock burns were not affected. Tibia Zn content was higher in HCZ or ORZ-supplemented birds compared to NC (*P* < 0.05), whereas other minerals in tibia and liver were unaffected. In summary, the absence of supplemental Zn negatively affects growth and carcass characteristics, whereas replacing ZnSO_4_ with HCZ, ORZ, or their combinations improves bodyweight and FCR. HCZ70-ORZ10 and HCZ40-ORZ40 were identified as optimal combinations for maximizing feed efficiency.

## Introduction

Chicken meat remains the primary source of animal protein globally, both in developing and developed countries [1]. Genetic selection and precise nutrition have been crucial in enhancing meat chicken productivity [2, 3]. Today, meat chicken diets are balanced for more than 33 different nutrients including energy, amino acids, vitamins, macro minerals, and trace minerals [4]. Among the trace minerals, Zn assumes significant importance, primarily due to its involvement in various physiological functions and its essentiality as a micronutrient [5]. Zinc is a component of numerous enzymes involved in digestion, metabolism, and immune function [6, 7]. For example, Zn is crucial for the activity of enzymes that aid in protein and carbohydrate digestion, as well as for the synthesis of nucleic acids and proteins [8]. It supports the development and activity of immune cells, helping the birds to resist infections and maintain overall health [9]. Zinc deficiency can compromise the immune responses, making birds more susceptible to diseases [10]. Furthermore, Zn is involved in cellular division and differentiation, which are essential processes for growth and tissue repair [11]. Therefore, Zn is an essential trace mineral crucial for optimal growth and proper functioning of physiological processes within the body [12].

Historically, Zn has been supplemented in the form of inexpensive inorganic salts such as sulfate (ZnSO_4_) and oxide (ZnO) in broiler diets to meet the requirements. However, the ionic bonds in inorganic salts are very weak making these salts highly water soluble and thus reactive both in the feed and inside the body [13, 14]. Thus, Zn supplied in the form of inorganic salts may interact antagonistically with other minerals, such as Fe and Cu, vitamins, and enzymes, potentially reducing both Zn and its antagonist’s bioavailability and utilization by the birds [15]. Because of lower bioavailability and cheap price, higher quantities of Zn inorganic salts are added to the feed to prevent Zn deficiency, which could result in excess Zn excretion to the environment through litter and manure, posing risks to soil and water quality [16]. Therefore, there has been a growing interest in finding alternative sources of Zn that are more bioavailable and less likely to cause environmental issues, such as organic forms, and chelated or hydroxychloride minerals [17, 18]. Organic sources of Zn have been researched extensively and proposed as effective alternatives to inorganic forms [18, 19]. Replacing inorganic Zn with organic sources (Zn proteinate) has been reported to improve growth performance and intestinal barrier function under necrotic enteritis challenge [20], and intestinal morphology and oxidative stress in young broilers [21]. Farhadi Javid et al. [22] reported that zinc threonine has higher relative bioavailability compared to ZnSO_4_, as evidenced by improvements in body weight, feed conversion ratio, cholesterol levels, superoxide dismutase enzyme activity, and both ash and zinc content in the tibia of broiler chickens. However, despite the proven advantages of organic Zn over the inorganic sources in poultry production, its higher cost currently limits its widespread adoption and integration within the chicken meat industry [23].

Zinc hydroxychloride (HCZ), a relatively new class of Zn source for livestock, is structured through covalent bonds involving Zn atoms, multiple hydroxyl groups, and chloride ions, creating a robust chemical bond that surpasses the ionic forms [24]. This crystalline structure renders HCZ less soluble in water compared to its ionic counterparts, thereby minimizing the potential for antagonistic reactions in both feed and the upper digestive tract [25, 26]. Previous studies have reported improvements in growth, meat yield, and bone breaking strength when feeding equivalent levels of Zn from Zn hydroxychloride in comparison to ZnSO_4_ [27, 28]. For example, Chawalitjinda et al. [29] reported that replacing ZnSO_4_ with HCZ at 50 mg/kg improved body weight gain, reduced mortality, and enhanced zinc deposition in the liver of broiler chicks. Combining HCZ and copper hydroxychloride improved bone development and skin integrity in broiler chickens and was identified as an effective nutritional strategy to prevent leg disorders [30]. HCZ, though not categorized as an organic source of Zn, is relatively more economical compared to organic sources. Thus, combining hydroxychloride Zn with organic forms could present a cost-effective alternative to straight organic Zn in broiler feed. However, there is limited information on whether such combinations can yield synergistic effects or similar benefits while maintaining low production costs in broiler chicken farming. Therefore, this study aimed to assess the impact of combining organic Zn (Optimin®; Nutreco N.V., Amersfoort, Netherlands) and hydroxychloride Zn (IntelliBond®; Nutreco N.V., Amersfoort, Netherlands) at varying ratios compared to ZnSO_4_ on the growth performance, carcass characteristics, and mineral profiles of tibia and liver in broiler chickens reared to 35 days of age.

## Material and Methods

All the experimental procedures applied in this study were reviewed and approved by the University of New England Animal Ethics Committee (Authority No.: AEC19-017) to be according to the national and international guidelines.

### Experimental Design and Birds Management

A total of 952 1-day-old male Ross 308 broiler chicks were obtained from Aviagen hatchery (Goulbrun, NSW, Australia). The birds were vaccinated for Marek’s disease, infectious bronchitis, and Newcastle disease at the hatchery. Upon arrival, chicks were weighed and assigned to seven dietary treatments based on a completely randomized design. Each diet was replicated 8 times in floor pens with 17 chicks per replicate. The experimental diets were based on wheat, corn, soybean, and canola meals, and supplemented with 100 g/t xylanase and 500 FTU of phytase and formulated to precisely meet the nutrient specifications recommended by the breeder [4] (Table 1). The diets were offered in three phases: starter (0–10 days), grower (10–24 days), and finisher (24–35 days). The seven experimental diets included a negative control diet (NC) without any supplemental Zn, and six diets with 80 mg/kg added Zn in the forms of ZnSO_4_ (positive control; PC), hydroxychloride Zn (HCZ) and organic Zn (ORZ), and HCZ-ORZ combination at 40 mg/kg HCZ + 40 mg/kg ORZ (HCZ40-ORZ40), 55 mg/kg HCZ + 25 mg/kg ORZ (HCZ55-ORZ25), and 70 mg/kg HCZ + 10 mg/kg ORZ (HCZ70-ORZ10). The hydroxychloride Zn was from IntelliBond® (55% Zn content; Nutreco N.V., Amersfoort, Netherlands) and the organic Zn was from Optimin® (15% Zn content chelated to small peptides; Nutreco N.V., Amersfoort, Netherlands) sources. The mineral premixes were prepared in our nutrition lab, with each Zn source added at the specified levels to the premix. The inclusion rate of the mineral premix was maintained at a constant rate of 1.0 kg/t, using a blend of rice hulls and fine limestone as fillers across all formulations. Each premix was thoroughly mixed individually using a cake mixer, and sub-samples were collected for mineral analysis. All the diets were pelleted using a steam pellet machine, and the starter diet was further crumbled to maximize feed intake. The shed temperature was initially set and maintained at 34 ± 1.0 °C during the first 3 days and then gradually reduced to 23 °C by week 3 of the experiment. Lighting program and ventilation followed the recommendations set in the Ross 308 management guide [31]. Chicks were exposed to 23 h of light and 1 h of dark for the first 7 days. During the grower phase, dark hours increased to 4 h, and during the finisher phase, to 6 h. Light intensity was set at 40 lx for the first week, decreasing gradually to 10 lx by day 10 and remaining constant thereafter. Relative humidity was kept between 60 and 70% during the first week, decreasing to below 40% by day 12. Birds had ad libitum access to water and feed throughout the entire study.

**Table 1:** Experimental diets and their calculated nutrient composition

	Starter	Grower	Finisher
Ingredients %			
Wheat	33.1	30.1	34.1
Soybean meal	30.9	26.7	19.4
Corn	25.0	30.0	30.0
Canola meal	5.00	7.00	9.00
Canola oil	2.58	3.81	4.80
Limestone	0.88	0.84	0.82
Dicalcium phosphate^1^	1.04	0.89	0.73
Na bicarbonate	0.195	0.205	0.226
Lysine HCl	0.271	0.265	0.249
DL-methionine	0.286	0.242	0.171
Salt	0.245	0.215	0.169
L-threonine	0.134	0.117	0.090
Choline Cl 75%	0.050	0.050	0.050
Mineral premix^2^	0.100	0.100	0.100
Vitamin premix^3^	0.090	0.090	0.090
Xylanase	0.010	0.010	0.010
Phytase	0.005	0.005	0.005
Calculated nutrients^4^			
AMEn (kcal/kg)	3000	3100	3200
Crude protein	22.75	21.19	19.22
Crude fat	4.97	6.48	7.66
Crude fiber	3.66	3.71	3.68
Dig^5^ arginine	1.350	1.240	1.091
Dig lysine	1.260	1.162	1.024
Dig methionine	0.592	0.536	0.448
Dig Met + Cys	0.939	0.866	0.763
Dig tryptophan	0.252	0.231	0.206
Dig isoleucine	0.848	0.780	0.691
Dig threonine	0.845	0.780	0.687
Dig valine	0.945	0.881	0.800
Calcium	0.900	0.850	0.800
Phosphorus available	0.450	0.425	0.400
Phosphorus total	0.605	0.572	0.537
Sodium	0.200	0.190	0.180
Chloride	0.250	0.230	0.200
Choline (mg/kg)	1,700	1,600	1,500

^1^Dicalcium phosphate contained phosphorus, 18%, and calcium, 21% ^2^Trace mineral concentrate supplied per kilogram of diet: Zn (different sources), 80 mg; Fe (sulfate), 40 mg; I (iodide), 1.5 mg; Se (selenate), 0.3 mg; Mn (sulfate), 60 mg; millrun-based carrier, 128 mg; mineral oil, 100 mg ^3^Vitamin concentrate supplied per kilogram of diet: retinol, 12,000 IU; cholecalciferol, 5000 IU; tocopheryl acetate, 75 mg, menadione, 3 mg; thiamine, 3 mg; riboflavin, 8 mg; niacin, 55 mg; pantothenate, 13 mg; pyridoxine, 5 mg; folate, 2 mg; cyanocobalamin, 16 μg; biotin, 200 μg; cereal-based carrier, 149 mg; mineral oil, 2.5 mg ^4^Expressed as % unless specified otherwise ^5^*Dig*, digestible

### Data Collection

Birds’ body weights (BW) were recorded on days 1, 10, 24, and 35 post-hatch on a pen basis. Total BW of the pen and the number of birds in each pen were used to calculate the average BW and BW gain per bird at the end of each period. Feed intake was also recorded for the same intervals and used to calculate the feed conversion ratio (FCR). The BW of any dead or culled birds was recorded and used to correct the FCR. As there were treatment associated differences in body weights, the FCR was further corrected to the final BW of the birds in the PC group. Each 50 g difference in BW was considered to be equivalent to 0.01 of FCR.

On day 34 of the trial, all the birds within each pen were visually inspected and scored for the severity of foot pad lesions (dermatitis) and hock burns according to the method described by Nguyen et al. [32]. On day 35 of the trial, three birds per replicate pen, representing pens mean BW, were euthanized for sample collection. Breast meat, leg quarter (thigh + drumstick), abdominal fat pad, gizzard, and liver were removed and weighed. The relative weight of all the above was calculated and expressed as g/100 g of live BW. The whole liver was collected in plastic bags and frozen at − 20 °C and then freeze-dried. Tibias were removed and collected individually.

### Tibia Breaking Strength and Ash Percentage

The tibiae underwent breaking strength testing using an Instron instrument (LX300 Instron Universal Testing Machine, Instron Corp., Canton, Norwood, MA, USA) equipped with a 300 kN load cell and 3-point fixture bed, operating at a test speed of 10 data points per second and controlled by Blue Hill 3 software. Subsequently, the tibia bones were dried at 105 °C for 24 h in a drying oven (Qualtex Universal Series 2000, Watson Victor Ltd., Perth, Australia) to determine dry mass, followed by ashing in a chamber furnace (Carbolite CWF 1200, Sheffield, UK) at 600 °C for 6 h. The percentage of moisture-free tibia ash was calculated based on the dry weight of the tibia.

### Mineral Analysis

The mineral concentration in the diets, premixes, tibiae, and livers was determined using an inductively coupled plasma-optical emission spectrometer (ICP-OES) (Agilent, Mulgrave, Victoria, Australia). Representative composite samples from each diet and mineral premix (as-fed basis) were collected in triplicate and ground into fine particles (0.5 mm) for analysis of mineral concentration in duplicate. Subsequently, 0.5 g of each diet sample was placed into white Teflon tubes (Milestone, Sorisole, Bergamo, Italy) and digested in 1 mL distilled water and 4 mL concentrated HCl (70%) using an Ultra wave Microwave Digestion system (Milestone, Sorisole, Bergamo, Italy). The digested samples were then diluted with distilled water to a final volume of 25 mL in a 30-mL volumetric flask for subsequent analysis of mineral concentration using an ICP-OES instrument. For the tibia and liver, 0.1 g of each ash sample was weighed for analysis of mineral concentration by ICP-OES technique (as described above).

### Statistical Analysis

Prior to statistical analysis, all data were assessed for normal distribution. Analysis was performed using one-way ANOVA through the General Linear Model procedure in JMP®13 (SAS Institute Inc., JMP Software, Cary, NC). Each pen served as an experimental unit, and the values presented in the tables represent means with pooled standard error of the mean (SEM) (*n* = 56). Where a significant effect of treatments was detected (*P* ≤ 0.05), means were separated using Tukey’s test.

## Results

The analyzed mineral composition of the mineral premixes and experimental diets used in this study are presented in Tables 2 and 3, respectively. The analyzed Zn concentration in the NC premix and diet suggests that the intended supplemental Zn in both the premixes and the diets was most likely achieved. The concentration of the rest of the minerals was also very close, indicating proper mixability and even distribution of mineral premixes and other ingredients in the feed.

**Table 2:** Analyzed mineral composition of the premixes (performed in duplicate)

Premixes	g mineral/kg premix			
	Fe	Mn	Cu	Zn
Negative control (no Zn)	42.7	60.1	15.2	0.21
Positive control (ZnSO_4_)	43.1	60.7	15.4	81.1
HCZ 80 mg/kg	42.1	59.8	15.0	82.0
ORZ 80 mg/kg	42.6	60.2	14.8	81.6
HCZ 40 – ORZ 40 mg/kg	41.4	61.0	15.5	80.8
HCZ 55 – ORZ 25 mg/kg	43.0	61.1	15.2	80.2
HCZ 70 – ORZ 10 mg/kg	42.2	59.9	15.0	81.4

*HCZ*, hydroxychloride Zn from IntelliBond®; *ORZ*, organic Zn from Optimin®

**Table 3:** Analyzed mineral composition of the experimental diets for different phases

Diets	Mineral concentration					
	Ca %	P %	Fe µg/g	Mn µg/kg	Cu µg/g	Zn µg/g
Starter diets						
Negative control	0.86	0.66	175.7	110.6	25.0	44.1
Positive control	0.89	0.65	180.5	109.0	26.4	120.0
HCZ 80 mg/kg	0.88	0.65	177.5	106.4	24.8	123.7
ORZ 80 mg/kg	0.87	0.66	183.9	109.1	24.3	125.8
HCZ 40 – ORZ 40 mg/kg	0.87	0.64	175.3	107.1	25.2	122.1
HCZ 55 – ORZ 25 mg/kg	0.88	0.65	174.6	111.5	24.9	122.3
HCZ 70 – ORZ 10 mg/kg	0.90	0.64	186.6	108.4	25.0	125.5
Grower diets						
Negative control	0.85	0.61	167.5	97.6	27.6	46.3
Positive control	0.85	0.62	168.5	101.5	23.5	120.3
HCZ 80 mg/kg	0.86	0.61	169.4	103.2	27.9	125.7
ORZ 80 mg/kg	0.84	0.61	165.4	99.6	24.8	126.1
HCZ 40 – ORZ 40 mg/kg	0.86	0.61	173.8	105.8	26.1	125.1
HCZ 55 – ORZ 25 mg/kg	0.83	0.63	166.8	97.4	28.2	124.0
HCZ 70 – ORZ 10 mg/kg	0.84	0.61	171.5	102.6	27.5	126.9
Finisher diets						
Negative control	0.77	0.57	145.0	108.7	22.9	39.2
Positive control	0.79	0.59	153.5	106.1	24.8	116.0
HCZ 80 mg/kg	0.80	0.60	156.6	114.8	19.1	126.8
ORZ 80 mg/kg	0.80	0.60	155.5	115.9	22.7	122.7
HCZ 40 – ORZ 40 mg/kg	0.77	0.61	162.4	107.1	21.8	123.5
HCZ 55 – ORZ 25 mg/kg	0.80	0.58	160.1	110.6	26.0	126.2
HCZ 70 – ORZ 10 mg/kg	0.79	0.57	153.3	109.8	24.6	120.2

*HCZ*, hydroxychloride Zn from IntelliBond®; *ORZ*, organic Zn from Optimin®

Table 4 summarizes the effect of dietary treatments on growth performance over different phases and the entire production period of 35 days. Birds in the NC group had the lowest BW and highest FCR on day 10 compared to all the other treatments (*P* < 0.05). There was no significant effect of supplemental Zn source on BW or BWG over the starter phase (*P* > 0.05). However, the inclusion of HCZ improved FCR by almost 4 points compared to the PC with ZnSO_4_ (*P* < 0.05). BW on day 24 was statistically similar between NC and PC, but birds in all HCZ and ORZ treatments had a significantly higher BW compared to NC birds (*P* < 0.05). Over the grower period, the highest FCR was observed in NC groups followed by PC, and supplementation of ORZ at 80 mg/kg and a combination of HCZ at 40 mg/kg + ORZ at 40 mg/kg significantly improved FCR compared to both NC and PC groups (*P* < 0.05). The highest BW on day 35 was recorded for birds in HCZ70 + ORZ10, and HCZ40 + ORZ40 treatments (*P* < 0.05). Having HCZ and ORZ or their combination improved FCR by approx. 6.0 and 4.0 points compared to NC and PC treatments, respectively, during the finisher period (24–35 d; *P* < 0.05). Considering the entire production period (1–35 days), birds in all HCZ and ORZ treatments had a significantly better FCR compared to PC birds (*P* < 0.05), and the combination of 40 mg/kg HCZ and 40 mg/kg ORZ resulted in the lowest FCR. Correcting the FCR values for the BW of birds in PC treatments made HCZ40-ORZ40 and HCZ70-ORZ10 to exhibit the most efficient feed conversion compared to PC (1.418 vs 1.364, 1.366). There was no significant effect of dietary treatments on feed intake for the starter, grower, or finisher phases (*P* > 0.05). However, HCZ at 80 mg/kg and HCZ70-ORZ10 tended (*P* = 0.059) to increase feed intake over the grower period. Liveability rate (1–35 days) was not significantly affected by the treatments (*P* > 0.05).

**Table 4:** Performance parameters of broiler chickens in response to dietary treatments over different phases of growth

Treatments^1^	NC	PC	HCZ 80	ORZ 80	HCZ 40 ORZ 40	HCZ 55 ORZ 25	HCZ 70 ORZ 10	SEM	***P***-value
BW (g/b)^2^									
Day 1	37.8	37.7	37.5	37.7	38.1	37.8	37.9	0.207	0.493
Day 10	271^b^	284^a^	283^a^	280^a^	289^a^	282^a^	281^a^	3.141	0.021
Day 24	1313^b^	1345^ab^	1371^a^	1380^a^	1362^a^	1368^a^	1384^a^	10.73	0.003
Day 35	2511^c^	2548^bc^	2583^ab^	2592^ab^	2610^a^	2578^ab^	2623^a^	16.61	0.003
BWG (g/b)									
D 1–10	233 ^b^	247 ^a^	245 ^a^	242 ^a^	250 ^a^	244 ^a^	242 ^a^	3.13	0.022
D 10–24	1045^c^	1060^bc^	1088^ab^	1099^ab^	1074^abc^	1085^ab^	1105^a^	9.95	0.005
D 24–35	1198	1202	1213	1212	1247	1210	1238	16.06	0.257
D 1–35	2473^c^	2510^bc^	2546^ab^	2554^ab^	2573^ab^	2540^ab^	2586^a^	16.59	0.003
FI (g/b)									
D 1–10	259	265	254	258	262	261	254	3.671	0.258
D 10–24	1408	1427	1440	1437	1402	1426	1457	12.84	0.059
D 24–35	1885	1866	1836	1832	1878	1836	1862	25.77	0.650
D 1–35	3552	3559	3530	3528	3541	3524	3573	28.11	0.862
FCR (g/g)									
D 1–10	1.109^a^	1.076^b^	1.037^c^	1.064^bc^	1.049^bc^	1.068^bc^	1.049^bc^	0.007	< 0.001
D 10–24	1.352^a^	1.346^ab^	1.323^abc^	1.308^c^	1.304^c^	1.314^bc^	1.319^abc^	0.008	0.003
D 24–35	1.574^a^	1.552^ab^	1.515^c^	1.511^c^	1.505^c^	1.517^bc^	1.503^c^	0.008	< 0.001
D 1–35	1.436^a^	1.418^a^	1.387^b^	1.381^b^	1.376^b^	1.387^b^	1.382^b^	0.005	< 0.001
BWc^3^ 1–35	1.443^a^	1.418^a^	1.380^b^	1.372^b^	1.364^b^	1.381^b^	1.366^b^	0.006	< 0.001
**Liveability %** (1–35 d)	96.1	96.9	97.6	96.1	98.4	97.6	98.4	1.282	0.728

^a–c^Values in a row with no common superscripts differ significantly (*P* ≤ 0.05)—Tukey test ^1^*NC*, negative control – no supplemental Zn; *PC*, positive control – 80 mg/kg Zn added in form of ZnSO_4_; *HCZ 80*, hydroxychloride Zn at 80 mg/kg; *ORZ*, organic Zn at 80 mg/kg; *HCZ 40 ORZ 40*, 40 mg Zn as HCZ and 40 mg Zn as ORZ; *HCZ 55 ORZ 25*, 55 mg Zn as HCZ and 25 mg Zn as ORZ; *HCZ 70 ORZ 10*, 70 mg Zn as HCZ and 10 mg Zn as ORZ ^2^Grams per bird ^3^FCR values corrected to the mean body weight gain (1–35 d) of PC group—1 point of FCR considered for every 50 g difference in BWG

According to the data presented in Table 5, feeding the NC diet significantly (*P* < 0.05) reduced breast meat yield compared to all the other treatments except PC and HCZ40-ORZ40. Although there was no significant (*P* > 0.05) effect of the Zn source on leg quarter yield, the values recorded for HCZ40-ORZ40, and HCZ55-ORZ25 treatments tended (*P* = 0.099) to be higher than other treatments. The birds in NC had the highest abdominal fat pad percentage while HCZ80 and HCZ70-ORZ10 groups had the lowest (*P* < 0.05). No statistically significant (*P* > 0.05) impact of dietary treatments was detected on liver and gizzard relative weight, tibia breaking strength, footpad dermatitis, and hock burn scores recorded on day 35.

**Table 5:** Carcass characteristics (g/100 g live weight), tibia breaking strength (N/mm^2^), foot pad, and hock lesions in response to dietary treatments recorded on day 35

Treatemnts^1^	NC	PC	HCZ 80	ORZ 80	HCZ 40 ORZ 40	HCZ 55 ORZ 25	HCZ 70 ORZ 10	SEM	*P*-value
Breast meat	16.7^b^	17.2^ab^	17.9^a^	17.9^a^	17.5^ab^	17.8^a^	17.6^a^	0.197	0.003
Leg quarter	18.6	18.5	18.4	18.9	19.2	19.3	18.7	0.236	0.099
Fat	1.11^a^	0.92^b^	0.75^c^	0.89^bc^	0.94^b^	0.95^b^	0.84^bc^	0.036	0.001
Liver	2.81	2.86	2.81	2.80	2.76	2.78	2.91	0.092	0.929
Gizzard	2.03	2.00	1.94	1.95	1.87	1.89	1.97	0.061	0.545
TB^2^	304	312	318	311	318	307	319	9.381	0.866
FPD^3^	1.46	1.32	1.32	1.31	1.26	1.31	1.18	0.314	0.998
HB^4^	1.72	1.88	2.07	1.97	1.75	1.37	1.97	0.283	0.663

Mean values are based on 3 birds per replicate and 8 replicates per treatment ^a–c^Values in a row with no common superscripts differ significantly (*P* ≤ 0.05)—Tukey test ^1^*NC*, negative control – no supplemental Zn; *PC*, positive control – 80 mg/kg Zn added in form of ZnSO4; *HCZ 80*, hydroxychloride Zn at 80 mg/kg; *ORZ*, organic Zn at 80 mg/kg; *HCZ 40 ORZ 40*, 40 mg Zn as HCZ and 40 mg Zn as ORZ; *HCZ 55 ORZ 25*, 55 mg Zn as HCZ and 25 mg Zn as ORZ; *HCZ 70 ORZ 10*, 70 mg Zn as HCZ and 10 mg Zn as ORZ ^2^*TB*, tibia breaking strength ^3^*FPD*, footpad dermatitis ^4^*HB*, hock burn

There was no statistically significant (*P* > 0.05) effect of dietary treatments on tibia ash and mineral uptake except for Zn. The lowest tibia Zn content was determined in the NC group (*P* < 0.05) compared to HCZ and ORZ treatments, but not significantly different from that of PC birds (Table 6).

**Table 6:** Tibia mineral deposition in response to dietary treatments determined on day 35

Treatments^1^	NC	PC	HCZ 80	ORZ 80	HCZ 40 ORZ 40	HCZ 55 ORZ 25	HCZ 70 ORZ 10	SEM	*P*-value
Ash (%)	44.5	44.2	44.6	44.7	43.9	44.3	45.5	0.359	0.110
Ca (%)	37.1	37.9	37.9	38.2	37.7	37.8	38.0	0.538	0.809
P (%)	17.6	17.9	18.0	18.2	17.9	17.9	17.8	0.217	0.736
Cu (µg/g)	0.45	0.77	0.33	0.55	0.36	0.26	0.31	0.191	0.527
Mn (µg/g)	9.51	9.34	9.12	9.72	9.10	9.32	8.94	0.421	0.875
Fe (µg/g)	311	303	325	321	304	301	295	12.45	0.602
Zn (µg/g)	338^b^	377^ab^	395^a^	401^a^	411^a^	389^a^	395^a^	10.14	0.002

Mean values are based on 3 birds per replicate and 8 replicates per treatment ^a–c^Values in a row with no common superscripts differ significantly (*P* ≤ 0.05)—Tukey test ^1^*NC*, negative control – no supplemental Zn; *PC*, positive control – 80 mg/kg Zn added in form of ZnSO4; *HCZ 80*, hydroxychloride Zn at 80 mg/kg; *ORZ*, organic Zn at 80 mg/kg; *HCZ 40 ORZ 40*, 40 mg Zn as HCZ and 40 mg Zn as ORZ; *HCZ 55 ORZ 25*, 55 mg Zn as HCZ and 25 mg Zn as ORZ; *HCZ 70 ORZ 10*, 70 mg Zn as HCZ and 10 mg Zn as ORZ

According to the data presented in Table 7, liver mineral content was not significantly affected by the dietary treatments (*P* > 0.05).

**Table 7:** Liver mineral deposition in response to dietary treatments determined on day 35

Treatments^1^	NC	PC	HCZ 80	ORZ 80	HCZ 40 ORZ 40	HCZ 55 ORZ 25	HCZ 70 ORZ 10	SEM	*P*-value
Ca (µg/g)	167	146	155	153	158	155	157	4.642	0.110
P (mg/g)	11.5	11.3	11.5	11.3	11.7	11.1	11.9	0.021	0.139
Cu (µg/g)	11.8	11.4	11.4	11.2	11.2	11.2	11.4	0.282	0.227
Mn (µg/g)	11.4	10.7	11.4	10.8	11.7	10.8	10.9	0.394	0.472
Fe (µg/g)	762	686	675	668	767	655	682	51.36	0.581
Zn (µg/g)	85.5	84.4	88.4	85.5	84.8	85.3	87.5	1.897	0.716

Mean values are based on 3 birds per replicate and 8 replicates per treatment ^a–c^Values in a row with no common superscripts differ significantly (*P* ≤ 0.05)—Tukey test ^1^*NC*, negative control – no supplemental Zn; *PC*, positive control – 80 mg/kg Zn added in form of ZnSO4; *HCZ 80*, hydroxychloride Zn at 80 mg/kg; *ORZ*, organic Zn at 80 mg/kg; *HCZ 40 ORZ 40*, 40 mg Zn as HCZ and 40 mg Zn as ORZ; *HCZ 55 ORZ 25*, 55 mg Zn as HCZ and 25 mg Zn as ORZ; *HCZ 70 ORZ 10*, 70 mg Zn as HCZ and 10 mg Zn as ORZ

## Discussion

The analyzed Zn concentration in the premixes and finished feed confirms that the target level of having 80 mg/kg supplemental Zn per Kg of the experimental diets for each phase was likely achieved (Tables 2 and 3). For many years, the NRC [33] served as the primary reference for poultry nutritionists and researchers in formulating balanced diets for broiler chickens and laying hens. However, since the mid-2000s, breeder companies have started publishing their own recommended nutrient specifications tailored to specific commercial breeds and strains of birds [4]. The NRC [33] recommended Zn level for broiler chickens is 40 mg/kg of feed. The average analyzed Zn concentration in the NC diets, coming from the background ingredients, was around 40 mg/kg, meeting the minimum requirements recommended by NRC. All the birds in the current study outperformed their genetic potential and gained higher weight and exhibited comparable feed conversion ratios compared to breeder guidelines [4], even the birds fed the NC diets without any supplemental Zn. These findings indicate that feeding a diet without supplemental Zn does not significantly impair growth rate, cause mortalities, or compromise bone integrity. However, such diets may affect the bird’s overall metabolism, potentially leading to reduced feed efficiency and increased abdominal fat deposition. Furthermore, under commercial settings, birds are usually exposed to more environmental stressors and encounter more disease challenges which may make them more susceptible to Zn deficiency. For example, in the current study, overall mortality remained below 3.0%, whereas in commercial scenarios, end-of-batch mortality typically exceeds 5% or higher.

The improved feed conversion ratio and higher final body weight of the birds fed the diets with either HCZ, ORZ, or their combinations compared to both negative and positive control birds indicate that fast growing modern strains of broiler chickens have higher requirements for Zn and will benefit from supplemental Zn of higher bioavailability compared to ZnSO_4_. These results are consistent with published literature indicating improved growth performance in broiler chickens fed diets supplemented with either organic or hydroxychloride Zn compared to inorganic Zn [21, 34]. Bones serve as a functional reserve of Zn in the body, and the Zn content in the tibia of broiler chickens has been recognized as a sensitive indicator of their response to Zn supplementation [35]. In the current study, tibia Zn concentration confirms higher bioavailability of Zn from HCZ and ORZ and their combinations. Similarly, Smiricky-Tjardes et al. [36] reported higher relative bioavailability (RVB) of hydroxychloride Zn compared to feed grade ZnSO_4_ in broiler chicks based on tibia Zn content. Bruerton [37] reported a 35% increase in tibia Zn content in broilers fed chelated Zn when compared to ZnSO_4_. Yu et al. [38] measured the RVB of HCZ compared to ZnSO_4_ using various response criteria in broiler chickens. They reported an RVB of 110.8% based on body weight gain and an RVB of 108 to 119% based on tibia Zn content, plasma alkaline phosphatase activity, CuZn superoxide dismutase levels, and pancreatic metallothionein mRNA expression. In line with the findings of the current study, other researchers have also reported improved performance metrics in broiler chickens fed diets supplemented with HCZ [28, 39], and organic Zn compared to ZnSO_4_ [10, 40]. In addition to its role in numerous metabolic and biochemical pathways, body Zn status also affects gut integrity, immune responses, and morphometric parameters, all of which can influence nutrient absorption and partitioning. Bortoluzzi et al. [20] reported the beneficial effect of Zn proteinate (organic Zn) in attenuating the intestinal inflammation and improved intestinal barrier function. Improved bone mineralization [41], enhanced antioxidant capacity [21, 22], and greater villus length [21] have been reported as responses to replacing ZnSO_4_ with either organic Zn or HCZ sources in poultry diets. However, to the best of our knowledge, no studies have reported the synergistic effect between HCZ and organic Zn observed in this study. In fact, birds fed the diets with either HCZ40-ORZ40 or HCZ70-ORZ10 exhibited the best feed efficiency corrected for BW. The synergistic effect observed between hydroxychloride Zn and organic Zn could likely arise from enhanced bioavailability, complementary absorption pathways, improved tissue distribution, and synergistic metabolic effects, all of which contribute to better growth performance outcomes. The concept of bioavailability in the context of trace minerals like Zn involves more than just absorption from the diet. It encompasses the entire process from absorption through to utilization within the body for specific physiological functions [42].

Birds in the NC group recorded the highest abdominal fat pad weight and the lowest breast meat yield. Consistent with current observations, previously published data also indicate that dietary Zn sources and levels in broiler chickens affect fat deposition and carcass yield components [43–45]. Zinc is essential for the activity of enzymes involved in lipid metabolism, including lipases and fatty acid synthases [46]. Without sufficient Zn, these enzymes may not function optimally, leading to decreased lipolysis and increased lipogenesis of fats. This imbalance can result in the accumulation of triglycerides and fatty acids in adipose tissue [47]. Furthermore, Zn is involved in insulin synthesis, secretion, and signaling [48]. Insulin plays a crucial role in regulating glucose and lipid metabolism by promoting glucose uptake into cells and inhibiting lipolysis in adipose tissue [49]. Zinc inadequacy can affect insulin optimal function, leading to impaired glucose metabolism and increased lipolysis, which contributes to elevated fat deposition [50]. In addition, impaired glucose uptake affects energy availability [51] which can decrease protein accretion leading to reduced growth rate and lower breast meat yield as observed in NC birds.

Liver mineral content in broiler chickens serves as an essential indicator of mineral metabolism reflecting the intake and absorption of essential minerals. The liver plays a vital role in Zn metabolism, acting as both a storage depot for this element and a site where various Zn-dependent proteins are synthesized [10]. In the current study, supplemental Zn or its source in the diet did not affect liver Zn or other minerals content. This finding suggests that despite the higher bioavailability of HCZ and ORZ, as well as their combination, the absorbed Zn was utilized in the body to support various metabolic and physiological processes mediated by Zn, rather than being stored in the liver as surplus Zn. Similarly, Zhang et al. [52] did not observe any changes in liver Zn content in broiler chickens fed diets supplemented with Zn levels ranging from 0 to 120 mg/kg. In contrast, other studies have demonstrated a linear increase in Zn content in the liver and kidneys of broilers in response to dietary Zn supplementation [45, 53]. However, the supplemental Zn used in the above studies were 0–320 [53] and 0–1500 mg/kg [45] which were significantly higher than the levels tested in the current study. In another study, Jarosz et al. [10] reported higher liver Zn in response to diet supplementation with Zn glycinate at 100 mg/kg of the feed compared to the negative control treatment without any supplemental Zn. Nonetheless, it is worth noting that the negative control diet in their study only had 21.6 to 23.1 mg/kg Zn, whereas in our study the Zn content in the NC diet was nearly doubled at 40 mg/kg. The liver Zn content, alongside performance results obtained in current study, highlights the importance of evaluating not just the bioavailability but also the metabolic utilization of Zn when assessing the nutritional benefits of various Zn sources in broiler diets.

## Conclusions

In summary, the results obtained in the current study indicate inadequate dietary Zn impairs growth and nutrient utilization in broiler chickens, leading to lower muscle accretion (breast meat) and increased abdominal fat deposition. Therefore, high-yielding broilers require more than the NRC-recommended 40 mg/kg/kg of feed due to their fast growth and turnover rates. Supplementary Zn from hydroxychloride, organic sources, or their combination is more bioavailable and improves performance and carcass characteristics compared to ZnSO_4_. The combination of hydroxychloride Zn and organic Zn enhances feed conversion ratio and body weight, with optimal results seen in diets containing 70 mg/kg HCZ + 10 mg/kg ORZ and 40 mg/kg HCZ + 40 mg/kg ORZ, warranting further research into Zn digestion and absorption.

## Data Availability

No datasets were generated or analysed during the current study.
